# Anti-Aging Potential of Plants of the Anak Dalam Tribe, Jambi, Indonesia

**DOI:** 10.3390/ph16091300

**Published:** 2023-09-14

**Authors:** Uce Lestari, Muhaimin Muhaimin, Anis Yohana Chaerunisaa, Wawan Sujarwo

**Affiliations:** 1 Doctoral Program, Faculty of Pharmacy, Universitas Padjadjaran, Sumedang 45363, Indonesia; 2 Department of Pharmacy, Faculty of Medicine and Health Sciences, Universitas Jambi, Jambi 36361, Indonesia; 3 Department of Biological Pharmacy, Faculty of Pharmacy, Universitas Padjadjaran, Sumedang 45363, Indonesia; 4 Center of Herbal Study, Universitas Padjadjaran, Sumedang 45363, Indonesia; 5 Department of Pharmaceutics and Pharmaceutical Technology, Faculty of Pharmacy, Universitas Padjadjaran, Sumedang 45363, Indonesia; 6 Ethnobotany Research Group, Research Center for Ecology and Ethnobiology, National Research and Innovation Agency (BRIN), Cibinong, Bogor 16911, Indonesia

**Keywords:** *Toona sinensis*, anti-aging agent, phytochemicals, Anak Dalam Tribe

## Abstract

The process of skin aging is a physiological phenomenon that can not be avoided. According to global population data, the rate of aging increases by approximately 13% every year. The impact of skin aging has become a significant concern and challenge for developed countries. Consequently, there has been a search for potential new anti-aging agents. This review aims to provide an overview of the current research status of plants of the Anak Dalam Tribe (Indonesian: *Suku Anak Dalam* [SAD]; referred to as SAD henceforth) in Jambi Province, Indonesia, for the development of potential new anti-aging agents. One such discovery is a product derived from natural ingredients with the ability to prevent premature aging. These new anti-aging plants have been used for centuries by the Anak Dalam tribe, for treating skin diseases and maintaining skin health through traditional remedies. Recent research on herbal formulations used by the SAD community in Indonesia for skin beauty treatments, reported by Research on Medicinal Plants and Herbs or RISTOJA, indicates that 64 plant species are used for skin care. Among these plants, *Toona sinensis*, *Curcuma heyneana*, *Curcuma zedoaria*, *Curcuma longa*, and *Kaempferia rotunda* are the most commonly used medicinal plants with anti-aging properties. *T. sinensis* is a tree, while the others are herbs. *T. sinensis* shows the highest potential for development as an anti-aging agent, with its extracts, active fractions, and bioactive quercetin isolates known to possess strong anti-aging activities both in vitro and in vivo. Furthermore, *C. heyneana*, *C. longa*, *C. zedoaria*, and *K. rotunda* also show potential for further research, and three of them have demonstrated good potential for in vivo anti-aging activities. Only *K. rotunda* demonstrates relatively weaker antioxidant activity compared to *T. sinensis*, *C. heyneana*, *C. longa*, and *C. zedoaria*. Nevertheless, *K. rotunda* can still be developed to search for potential opportunities as agents with other activities, while *T. sinensis*, *C. heyneana*, *C. longa*, and *C. zedoaria* in the findings could be an opportunity to explore the potential of new anti-aging agents. In conclusion, of the five medicinal plants traditionally used by the SAD in Jambi, Indonesia, *C. longa* has received the most extensive research and shows potential for the development of anti-aging solutions. *C. zedoaria*, *C. heyneana* and *K. rotunda* show good potential for in vivo anti-aging activity. *T. sinensis* is the least-studied medicinal plant. Nevertheless, it has potential for development, as it is widely used by the SAD community for both traditional medicine and skin care.

## 1. Introduction

The process of skin aging is a physiological phenomenon that cannot be avoided. According to the World Health Organization (WHO), approximately 72% of men and 42% of women under the age of 30 experience photoaging or premature aging. Researchers in Australia have observed an increasing trend since 1950, with approximately 8% of the global population experiencing aging, and this percentage continues to rise by 13% annually as of 2020 [1]. Indonesia, Malaysia, and the Philippines are three populous countries in Southeast Asia with a combined population of approximately 400 million, facing the issue of premature aging. Indonesia, in particular, is witnessing continuous growth in its aging population, with an estimated annual increase projected to reach 20% by 2050 [2]. The impact of skin aging has become a significant concern, greatly affecting the lives of modern society and presenting a major challenge for developed nations in addressing this issue [1]. For these reasons, to overcome aging, discoveries of potential new anti-aging agents have emerged. The importance of anti-aging agents includes preventing skin damage, reducing signs of aging, improving skin texture, improving skin tone, stimulating collagen and elastin production, preventing future skin problems, increasing self-confidence, reducing the risk of disease, and skin and holistic care [3]. 

Basically women worldwide typically take care of their skin by using cleansers containing oil or water, followed by the application of skincare products tailored to their specific skin conditions [4]. In Indonesia, women traditionally maintain their skin’s health and beauty by harnessing the power of spices derived from natural ingredients, while internal skincare is achieved through the consumption of herbal drinks [5,6]. This practice originates from ancestral customs, particularly among the Anak Dalam tribe (Indonesian: *Suku Anak Dalam* [SAD]; referred to as SAD henceforth) in Jambi Province, Indonesia. During wedding rituals, SAD women would traditionally apply a crushed mixture of plant roots all over their bodies, aiming to enhance their skin’s beauty and prevent aging [7].

The aging process occurs due to skin degeneration caused by oxidative stress in the body resulting from free radicals. Skin damage caused by free radicals affects biomolecules, such as lipids, proteins (enzymes), and nucleic acids, which are triggered by Reactive Oxygen Species (ROS) [1]. These ROS may lead to telomere shortening, autophagy, and exhaustion of stem cells, resulting in a decrease in collagen, elastin, and hyaluronic acid levels, collectively known as the extracellular matrix (ECM) [8,9,10]. Insufficient levels of ECM can cause loss of skin elasticity, the formation of fine lines and wrinkles, the appearance of dark spots, and a dull and rough skin texture [11]. Therefore, antioxidants can be utilized to reduce oxidative stress levels.

Antioxidants can be produced by the body itself to counteract free radicals through cellular oxidation reactions, but the body relies more on exogenous antioxidants, which can be either natural or synthetic. Natural antioxidants come from natural ingredients, such as fruits, vegetables, grains and plants. Examples of natural antioxidants include vitamin C (ascorbate), vitamin E (tocopherol), beta-carotene, and polyphenols found in green tea or red wine. Synthetic antioxidants are chemical compounds prepared in laboratories. They do not occur naturally in food [12,13]. Long-term use of synthetic antioxidants has been associated with potential carcinogenic effects, whereas natural antioxidants are abundantly available in nature without side effects. Furthermore, natural antioxidants are relatively safer and more cost-effective than synthetic antioxidants [14,15,16].

The use of natural ingredients as antioxidants has been a traditional skincare practice passed down through generations [17,18,19]. These natural antioxidants are derived from phytochemical sources obtained through extraction and isolation from herbal plants that grow in our surroundings [20,21,22]. Common phytochemical sources found in plants with natural antioxidant activity include phenolic compounds, polyphenols, flavonoids, cinnamic acid derivatives, coumarins, curcumin, tocopherols, and other polyfunctional organic acids [23,24].

Phytochemicals present in plants have played a significant role in cosmetic development for centuries, including the recent advancement of anti-aging agents. Compounds such as quercitrin (quercetin 3-rhamnosyl) and quercetin glycosides found in plants have antioxidant and anti-aging properties pharmacologically [25]. Indonesia, as the country with the second-highest number of native medicinal plants after the Amazon rainforest, holds great potential as a source of new anti-aging remedies. However, this potential has not been fully tapped into by the Indonesian people [22,26,27]. Despite that, the SAD residing in the forests of the Bukit Dua Belas region in Jambi Province, Indonesia has extensively utilized forest plants for medicinal purposes and as beauty treatments during traditional wedding rituals, particularly for SAD women [5,6,7,28]. Thus, this review highlights the current research status of these plants for the development of promising new anti-aging interventions. *C. heyneana*, *C. longa*, *C. zedoaria* and *T. sinensis* have very strong antioxidant activity so that their ability is synergistic in neutralizing ROS that cause aging and provide a great opportunity to be developed into potential new anti-aging compounds containing new active compounds, which is found in the plants *C. heyneana*, *C. longa*, *C. zedoaria* and *T. sinensis*.

## 2. Methods

**Figure 1:**
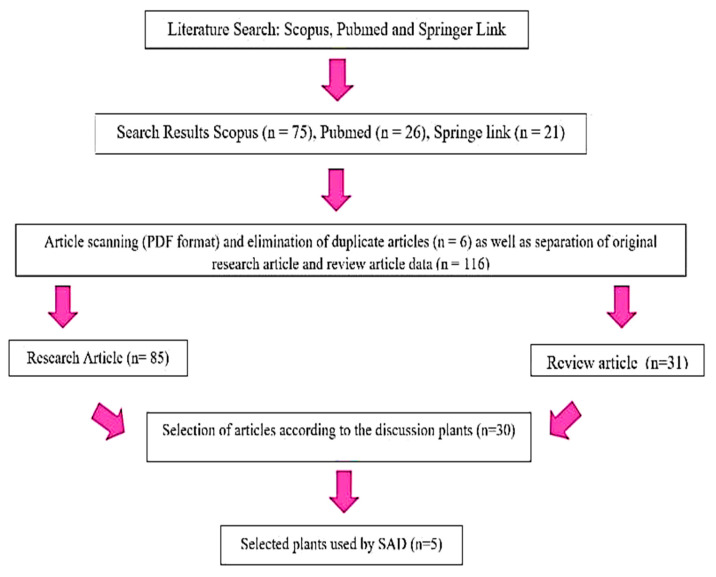
Flow diagram of this study.

The review was conducted in May 2023 and involved several steps. Firstly, a literature search was performed using Scopus, PubMed, and Springer Link, with specific eligibility criteria and keywords. The collected articles were then converted into PDF format and duplicate articles were eliminated. After that, they were categorized into research articles and review articles. Next, relevant articles were selected based on the topic of interest. Finally, the chosen articles were included in the review. The flow diagram is shown in Figure 1.

## 3. Herbs with Anti-Aging Properties

Indonesia, located near the equator, boasts a rich abundance of natural ingredients with herbal anti-aging properties [29,30]. The country’s tropical climate, characterized by high rainfall and temperatures, provides ideal conditions for the robust growth of plants compared to other regions. Indonesia experiences two distinct seasons: the rainy season and the dry season [31,32,33]. These seasonal variations significantly impact the phytochemical composition and activity of plants. For example, chokeberry exhibits high levels of phenolic and flavonoid compounds, ranging from 9,710 mg/L to 11,093 mg/L, along with strong antioxidant activity of 12.9–14.6 mmol/L, classified as highly potent. These levels are observed when the plant is cultivated under conditions of high rainfall and temperatures, surpassing those grown under less favorable conditions [19,34]. This potent antioxidant activity works synergistically to counteract Reactive Oxygen Species (ROS) involved in the photoaging process, commonly known as anti-aging activity [25]. Indonesia, specifically the province of Jambi, is home to many plants with antioxidant and anti-aging properties, where people use many herbal plants to use as masks and scrubs for facial and body skin care.

Jambi Province, Indonesia is home to the SAD, a minority community residing in the Bukit Dua Belas Forest Area. The SAD community has a tradition of utilizing traditional medicine for various ailments and skin beauty treatments, which have been passed down from their ancestors. According to data from the Research on Medicinal Plants and Traditional Herbal Medicine (Indonesian: *Riset Tanaman Obat dan Jamu* [RISTOJA]) in 2015, the Bukit Dua Belas Forest Area, located in Sarolangun Bangko Regency, Bungo Tebo Regency, and Batanghari Regency of Jambi Province, is home to 182 plant species used in herbal remedies for skin care. However, among these, only five plant species are specifically utilized by the SAD community for their skincare treatments [35,36]. The literature review reveals that only 30 plant species have been identified for their anti-aging properties.

Based on the study above, the Table 1 below presents the data on plants with anti-aging activities that are abundant in the Bukit Dua Belas area of Jambi, Indonesia, and widely utilized by the SAD community.

The literature review identified 30 plant species with anti-aging activity. Ethnobotanically, the plant families that are widely used to maintain the immune system and skin health are Zingiberaceae, Meliaceae, Ranunculaceae, Arecaceae, Muntingiaceae, Asteraceae, Myricaceae, Myrtaceae, Phylanthaceae, Apiaceae, Sapindaceae, and Rutaceae [39,40,41,42,43,44,49,50,51,52,55,56,57,62,64,65]. Meanwhile, ethnobotanically, the plant families that are not used to maintain the immune system and skin health but have anti-aging activity are Gnetaceae, Fabaceae, Simoroubaceae, Piperaceae, and Moringaceae and these still have to be introduced to the public regarding their use as anti-aging agents. Several references show that all but of them are predominantly utilized as antioxidants and anti-aging agents by the SAD community in Jambi, Indonesia [38,41,46,47,48,53,55,58,59,60,61,63,66]. These plants belong to the Zingiberaceae and Meliaceae families. Among them are *C. longa*, *C. heyneana*, *K. rotunda*, and *C. zedoaria* from the Zingiberaceae families, as well as *Toona sinensis* (*surian* leaves) from the Meliaceae family. Additionally, several plants from the Fabaceae family, including *P. indicus*, *P. santalinus*, *I. bijuga*, *G. max*, *S. littoralis and P. vulgaris* are also commonly used as antioxidants and anti-aging agents, although they are less frequently utilized by the SAD ethnic group [37]. This plant is only used as a cooking spice.

In general, each of the plant species above has both antioxidant and anti-aging activity, where this antioxidant activity works synergistically towards the ability to stabilize the role of Reactive Oxygen Species (ROS) in the photoaging process, so that it can have anti-aging activity by inhibiting the elastase enzyme and collagenase enzymes [67]. 

Antioxidants can be classified into two types: those derived from natural sources and those synthesized artificially. The long-term use of synthetic antioxidants can have adverse effects and may be carcinogenic. Therefore, there is a demand for natural antioxidant alternatives that are abundantly available in nature. Natural antioxidants work systemically and possess the ability to prevent aging in cells and tissues [14]. Examples of plants that meet these criteria include those from the Zingiberaceae family, such as *C. heyneana* and *C. longa*, which demonstrate antioxidant and anti-aging activities [68,69]. It can be seen that many SAD people use turmeric as a body scrub which is applied to the body at traditional wedding events for women, so that it can be developed into a new anti-aging potential from the curcumin compound contained in *C. longa*.

Based on the research conducted by Marianne et al. (2021) and Zeeshan et al. (2018), it has been found that the plant *C. heyneana* contains phytochemical compounds such as curcumin and demethoxycurcumin, which act as antioxidants [68,69]. In a study by Rohman et al. (2020), the antioxidant activity of curcumin extracted from *C. heyneana* was evaluated using the DPPH method, and it exhibited a strong antioxidant capacity with an IC_50_ value of 62.5 µg/mL [70]. This indicates that the potent antioxidant activity of curcumin works synergistically to stabilize Reactive Oxygen Species (ROS) and combat photoaging, commonly referred to as anti-aging activity [70]. *C. heyneana* is widely used by people for skin care by brewing and drinking it so that it can soften the skin from the inside against exposure to free radicals.

However, the therapeutic efficacy of curcumin as an antioxidant and anti-aging agent is still under clinical debate due to various factors. These include its poor bioavailability, rapid metabolism and elimination, low solubility, instability in gastric pH, presystemic metabolism in the liver, and absorption issues that lead to subtherapeutic plasma concentrations and reduced therapeutic effectiveness [30,31,32]. Currently, the latest development is nanodelivery systems for herbal anti-aging products as an approach to address the challenges associated with the direct use of pure phytochemicals in therapy [31,34,35,71]. There are still very few nanocosmetic preparations of natural origin such as curcumin as anti-aging agents.

*Curcuma heyneana* is rich in curcumin, which exhibits anti-aging properties and is extensively used by the SAD community. This plant is abundant in the Bukit Dua Belas area. Traditionally, *C. heyneana* is employed by the SAD community for skin care, specifically as a body scrub applied to the skin of SAD brides to enhance skin softness and eliminate unpleasant body odors. This scrub is sometimes prepared by combining *C. heyneana* with other ingredients such as rice flour, turmeric, pandan leaves, and lime, resulting in a natural scrub that is applied daily to the skin before SAD weddings [40,72]. The *C. heyneana* scrub indirectly acts as a natural antioxidant and synergistically prevents premature aging or anti-aging effects [70,73,74]. 

Furthermore, *C. longa* is another widely utilized plant in traditional herbal cosmetics, particularly as a face mask or scrub to smoothen the skin and remove dead skin cells. In the SAD community of Jambi, Indonesia, this plant is also consumed as a health drink called *jamu kunir asam* to improve skin texture because it contains antioxidant compounds that not only neutralize free radicals but also possess anti-aging properties [75,76].

The anti-aging activity of these plants is attributed to the curcumin—a phytochemical compound—which is present not only in *C. longa* and *C. heyneana* but also in other plants, such as *C. zedoaria* and *K. rotunda*. The research conducted by Desmiaty et al. (2018)—in which they conducted the antioxidant activity test using the DPPH method—indicated that *C. zedoaria* obtained an IC_50_ value of 2.24 ppm (indicating a very strong category). On the other hand, *K. rotunda* showed an IC_50_ value of 193.17 ppm (indicating a weak category). Additionally, *C. zedoaria* demonstrated an elastase inhibition capability of 49.24%, while *K. rotunda* exhibited an elastase inhibition capability of 40.82%. These findings indicate that the antioxidant activity of these plants is closely linked to their anti-aging effects [77,78,79]. *K. rotunda* has weaker antioxidant activity compared to *C. zedoria*, but the chemical compounds contained in *K. rotunda* can still be developed as potential agents for other activities such as antielastase, antithyrosinase, UV protection, antibacterial, antimutagenic, anticancer, antinociceptive, antihyperglycemic, anti allergic, anti-androgenic, anthelmintic, and wound healing [79].

Apart from *C. heyneana*, *C. longa* is also extensively used by the SAD community for herbal skincare treatments. It is applied as a face mask or scrub to smoothen the skin and remove dead skin cells, particularly for brides. *Curcuma longa* is not only employed as a herbal cosmetic but is also consumed as a health beverage by the SAD community to boost immune function. This plant contains antioxidant compounds that not only combat bacteria and reduce the risk of diseases but also act as a remedy for throat inflammation. Consequently, it is common to find that SAD people are shielded from illnesses and possess robust immune systems throughout their lives in the forest [76,80,81].

Based on their color, turmeric can be classified into different varieties, including yellow or red turmeric (known as *C. longa*), black turmeric, and white turmeric (known as *C. zedoaria*). The yellow and white turmeric varieties are known to possess anti-aging properties beneficial for skin care. *Curcuma zedoaria* (white turmeric), which is commonly found in Indonesia, particularly in the Bukit Dua Belas area, is frequently used by the SAD community for traditional remedies to treat various ailments. In addition to *C. zedoaria*, *K. rotunda* is also utilized in traditional medicine [77,81].

One of the plants from the Meliaceae family is *T. sinensis*, commonly referred to as the Chinese mahogany tree. According to interviews conducted with the SAD community, the leaves of this tree are extensively utilized for treating skin conditions such as itching. To apply it, the leaves are crushed and applied to the affected itchy skin. Furthermore, other parts of the tree, including the stem, bark, roots, and fruits, are commonly employed for the treatment of various ailments [36,82]. Many SAD people use surian stems as building material to make pillars for their houses, the bark is used as a diarrhea medicine and astringent, the roots are used as a detoxifier while the fruit is used to treat eye infections.

*Toona sinensis* is a plant commonly found in Indonesia and has the potential as a natural source of antioxidants. This plant is extensively used in traditional medicine for treating skin conditions, deworming, and dysentery, as an expectorant, tonic, blood sugar reducer, and for syphilis treatment [83]. The plant contains various secondary metabolites, including limonoids, phytol, flavonoids, essential oils, triterpenoids, phenols, and catechins [84], which possess the ability to counteract different pathological conditions in the body caused by oxidative stress related to aging and inflammation processes [85]. In the SAD community, many use surian leaves to treat skin diseases and also skin care, so that it can be developed into a potential new anti-aging agent from the new compounds discovered.

*Toona sinensis* is extensively utilized in Traditional Chinese Medicine (TCM), and its various parts, including the leaves, stems, bark, roots, and fruits, have been employed for treating a wide range of ailments. Traditionally, the stems and leaves of this plant are used for addressing conditions such as dysentery, enteritis, carminative effects, and skin disorders such as itching [82]. Furthermore, the roots are used as curatives, the bark is used as an astringent and depurative, and the fruits are utilized as an astringent for treating eye infections [36].

Therapeutic activities derived from natural substances have certain limitations, such as poor bioavailability, instability in gastric pH, low solubility, presystemic metabolism in the liver, and issues related to solubility and absorption within the body. These factors can lead to subtherapeutic plasma concentrations, resulting in a lack of therapeutic effects when using herbal remedies as medicines or cosmetics [32]. To address these limitations, the development of new nanotechnology-based approaches for herbal anti-aging is being pursued as a promising solution.

## 4. Phytochemistry

Recent research indicates the use of skin beauty treatments for anti-aging purposes among the SAD community in Indonesia. According to data from the Research on Medicinal Plants and Traditional Herbal Medicine (Indonesian: Riset Tanaman Obat dan Jamu [RISTOJA]) in 2015, it was found that 64 plant species have been employed for skin care. The most commonly used medicinal plants for anti-aging by the SAD community include *T. sinensis*, *C. heyneana*, *C. zedoaria*, *C. longa*, and *K. rotunda* [37].

*Toona sinensis*, known as Chinese mahogany, belongs to the Meliaceae family. Its local name is *surian merah*. It is a woody plant commonly found in East and Southeast Asian countries [86], including Indonesia. *Toona sinensis* is widespread in various regions of Indonesia, such as Sumatra, East Kalimantan, North Sulawesi, South Sulawesi, Maluku, Bali, West Nusa Tenggara, and Papua [87]. In Indonesia, Malaysia, and China, this plant is used as a vegetable source, while in India, it is used as animal feed [88].

Since the 1970s, sterol compounds have been discovered in the leaves of *T. sinensis* in China. Consequently, research has focused on studying the comprehensive phytochemical constituents of *T. sinensis*. To date, more than a hundred compounds have been isolated and identified from this plant, including terpenoids, phenylpropanoids, and flavonoids [74]. Flavonoids are commonly found in various plants worldwide. Thirteen flavonoids have been isolated and identified in different parts of this plant, including catechin, epicatechin, procyanidin B3, procyanidin B4, quercetin, quercitrin, isoquercitrin, rutin, kaempferol, rhamnopyranoside, astragalin, myricetin, and myricitrin [87,89,90]. The total purity of the flavonoids contained in *T. sinensis* is 66.60% to 71.05% [91]. Apart from flavonoids, the terpenoid group in *T. sinensis* has also been studied for its biological activity as an antioxidant and anti-aging agent. The results of the isolation and characterization of *T.sinensis* such as limonoids, apotirucallane, cycloartana [92]. The active compounds mentioned above such as quercetin, quercitrin, isoquercitrin have anti-aging activity.

**Figure 2:**
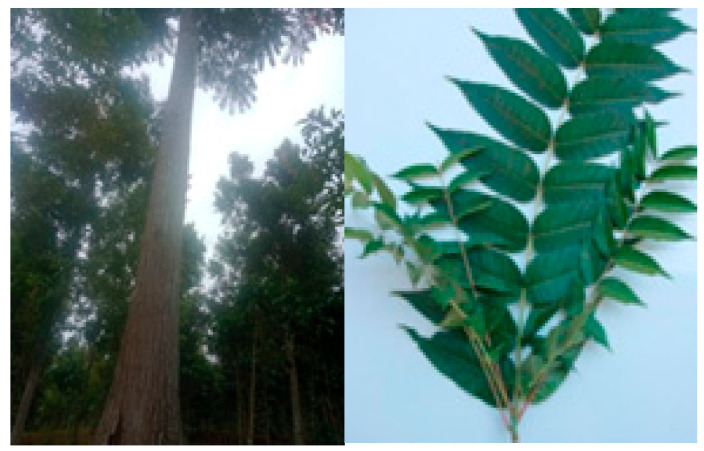
*Toona sinensis* [88].

Phytochemical screening of the *T. sinensis* plant has revealed the presence of terpenoids, phenylpropanoids, flavonoids, and anthraquinones. Previous research has reported that *T. sinensis* exhibits antitumor, antioxidant, antidiabetic, anti-inflammatory, antibacterial, and antiviral activities [91,93]. The leaves of this plant contain various bioactive compounds, such as gallic acid, methyl gallate, kaempferol, quercetin, rutin, quercitrin, palmitic acid, linoleic acid, and others [26]. Compounds such as quercitrin (quercetin 3-rhamnosyl) and quercetin glycosides are commonly found in the leaves of this plant. Pharmacological studies have shown that quercitrin possesses antioxidant, anti-inflammatory, and anti-allergic properties [26]. Figure 2, are some images of *T. sinensis*. Figure 2 is reprinted/adapted from Peng, W. et al. (2018) [88].

The surian plant has two types of leaves, namely red surian known as *T. sinensis* and green surian known as *T. sureni*. *T. sinensis* of the red surian species has a very high an-thocyanin and flavonol content compared to *T. sureni*. The content of this compound affects its biological activity as an antioxidant where *T. sinensis* of the red surian species has very strong antioxidant activity compared to *T. sureni*, so that the red surian type of *T. sinensis* is a valuable source of natural antioxidants and is more sought after and desirable than the green *T. sureni* [94].

**Figure 3:**
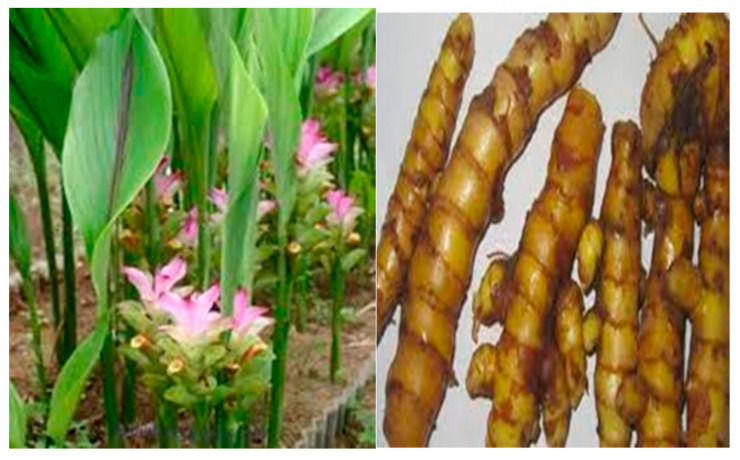
*Curcuma heyneana*.

Another plant that possesses anti-aging activity and contains bioactive compounds is *C. heyneana*, which is used to soften the skin and eliminate body odor due to its content of curcumin and its derivative, namely demethoxycurcumin [72]. The presence of curcumin in *C. heyneana* makes it widely utilized as an antioxidant and anti-aging agent [71]. Curcumin, which is found in *C. heyneana*, belongs to the flavonoid group of compounds and imparts a yellow color to the rhizome. Flavonoids are bioactive compounds commonly present in various parts of plants, including seeds, flowers, leaves, stems, and rhizomes. Flavonoids generally exhibit potent antioxidant activity, which is beneficial for the human body as they can repair damaged cells caused by free radicals and prevent premature aging [70]. The rhizome of *C. heyneana* is widely used in brewing drinks for skin care from within the body. Figure 3 shows *C. heyneana.*

According to a study conducted by Rohman, A. et al. (2020), through in vitro tests, such as the DPPH method for antioxidant activity, the extract of *C. heyneana* showed an IC_50_ value of 62.5 µg/mL. Additionally, the anti-aging activity was assessed by measuring the enzyme inhibition of tyrosinase and collagenase, resulting in an IC_50_ value of 31.25 µg/mL [40]. These findings suggest that the extract of *C. heyneana* is effective as an antioxidant and can combat free radicals on the skin exposed to sunlight. This can be attributed to the presence of bioactive phytochemical compounds, particularly curcumin, in *C. heyneana*. Furthermore, this plant also demonstrates potent anti-aging activity, as evidenced by an IC_50_ value of <50 ppm or 50 µg/mL [40]. So that the *C. heyneana* rhizome extract can be developed for its anti-aging potential into anti-aging cosmetic dosage form.

**Figure 4:**
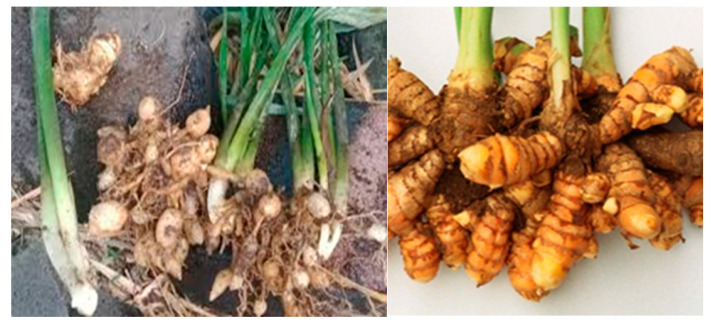
*Curcuma zedoaria* and *Curcuma longa* [40,95].

Other plants from the Zingiberaceae family widely used by the SAD community for skincare during traditional ceremonies are *C. zedoaria* and *C. longa*. The phytochemical compound contained in these plants is curcumin. Curcumin, a natural polyphenol extracted from *C. zedoaria* and *C. longa*, exhibits various biological activities, including antioxidant activity [40]. Figure 4 shows images of *C. zedoaria* and *C. longa.*

The phytochemical compounds present in *C. zedoaria* and *C. longa* are yellow pigments known as curcuminoids, which consist of curcumin, demethoxycurcumin, and bisdemethoxycurcumin [95,96]. Curcumin is a widely studied and common phytochemical component [40]. The difference in phytochemical compounds between *C. longa* and *C. zedoaria* lies in their curcumin content. *C. longa* has the highest curcumin content, ranging from 3% to 8%, compared to *C. zedoaria*, which contains only 0.1% curcumin. A higher curcumin content results in a greater amount of yellow pigment in turmeric, as seen in *C. longa* [75]. Other chemical constituents include phenylpropanoids and other phenolic components, as well as terpenes such as monoterpenes, sesquiterpenes, diterpenes, triterpenes, alkaloids, steroids, and fatty acids [97]. This phenolic component has anti-aging activity. 

*C. zedoaria* has been found to contain various primary and secondary metabolites. The main components of the plant include starch, curcumin, essential oil, and gum Arabic. The rhizome of the plant has been found to contain more than 10 sesquiterpenes, including curcumin, ethyl p-methoxycinnamate, β-turmerone, β-eudesmol, zingiberene, dihydrocurcumin, furanodiene, α-phellandrene, 1,8-cineole, β-elemene, and germacrone [67,68,98,99].

**Figure 5:**
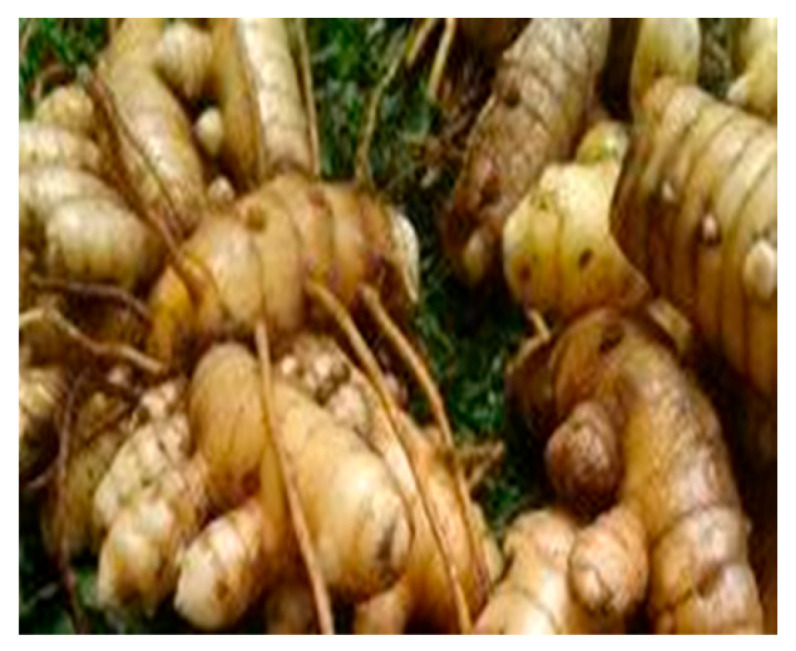
*Kaempferia rotunda*.

Curcumin is one of the most commonly used natural compounds as a potential modulator of cellular damage caused by free radicals. It is an active compound isolated from the plants *C. longa* and *C. zedoaria*, and it is also found in the plant *K. rotunda*. Curcumin exhibits antioxidant activity due to the presence of methylene hydrogen and phenol-o-methoxy groups [98]. Figure 5 shows images of *Kaempferia rotunda*. 

**Figure 6:**
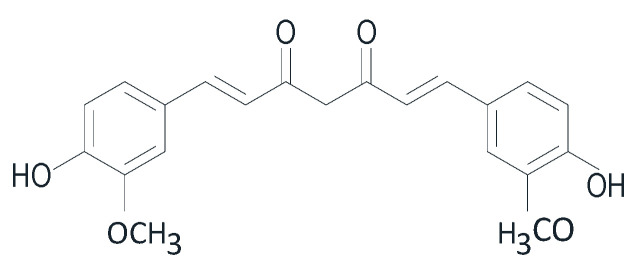
Chemical structure of curcumin.

However, curcumin’s therapeutic potential has limitations, such as its low bioavailability related to poor solubility, stability, and absorption in the digestive system. To address these issues, a study by Atarian, M. et al. (2019) utilized a nanotechnology-based system, specifically nanohydrogel curcumin with chitosan polymer, for the delivery of natural antioxidant therapy [100]. Despite that, the therapeutic efficacy of curcumin is challenged in clinical trials due to its low bioavailability and rapid metabolism and elimination [27]. Therefore, further development of curcumin compounds into nanoparticle formulations is necessary to improve their bioavailability. Figure 6 shows the chemical structure of curcumin.

The chemical structure of curcumin shown above, it reveals that curcumin contains methylene hydrogen and phenol-o-methoxy groups. It is derived from the isolation of *C. zedoaria* and *K. rotunda*. In vitro testing was conducted to examine its mechanism of action, specifically its ability to inhibit elastase activity. It was reported that curcumin can protect against aging by inhibiting elastase activity [39]. Many SAD people use *K. rotunda* as a traditional medicine by brewing it and drinking it for healthy skin. 

**Figure 7:**
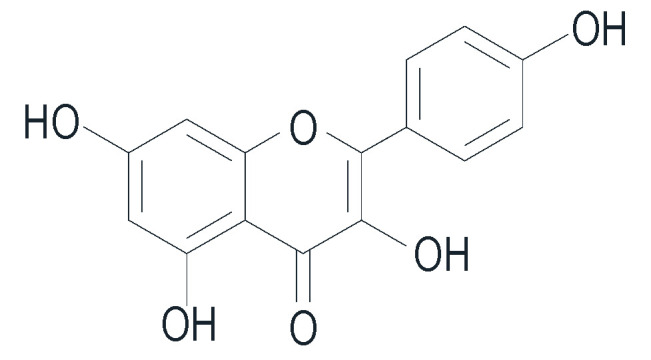
Chemical structure of kaempferol.

In addition to curcumin, *K. rotunda* also contains various chemical compounds. Previous studies have identified terpenoids, such as kaempulchraol and kaemgalangol, along with abietane, labdane, and clerodane types in this plant. It also contains phenolic compounds (diarylheptanoids, curcuminoids, and methoxylation derivatives of cinnamic acid), flavonoids (kaempferol and kaempferide), steroids and triterpenes (β-sitosterol), and essential oils (including ethyl trans-p-methoxycinnamate, ethyl cinnamate, cineole, camphene, borneol, pentadecane, and α-pinene) [101,102,103]. The characterization of the rutin of novel secondary metabolites with medicinal properties confirms the intra-specific variation among different *Kaempferia* species [104,105,106], highlighting unexplored curative potentials that require further detailed explanations. Kaempferol, which is present in *K. rotunda*, exhibits antioxidant activity that synergistically contributes to its anti-aging effects [106,107]. Figure 7 shows the chemical structure of kaempferol.

## 5. Pharmacological Activity

*T. sinensis* leaves with a soft texture not only have a delicious taste, but also have a very wide range of biological activities, one of which is as a very strong antioxidant activity [108]. *Toona sinensis* is widely used in Traditional Chinese Medicine (TCM), and various parts of this plant, such as leaves, stems, bark, roots, and fruits, have been used to treat various diseases. Traditionally, the stems and leaves of this plant are used for treating dysentery, and enteritis, as carminatives, and for alleviating itching and skin conditions [109]. The roots are used for curative purposes, the bark acts as an astringent and depurative, while the fruits are used as an astringent for treating eye infections [36]. Previous studies have reported that *T. sinensis* exhibits antitumor, antioxidant, antidiabetic, anti-inflammatory, antibacterial, and antiviral activities [36,93]. Pharmacological studies have shown that quercitrin possesses antioxidant, anti-inflammatory, and antiallergic properties [36].

**Figure 8:**
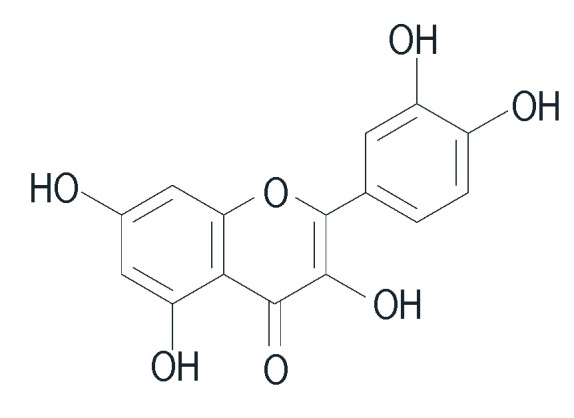
Chemical structure of quercetin.

Figure 8 shows the chemical structure of quercetin. Quercetin-3-O-α-L-rhamnopyranoside, isolated from *C. longa*, demonstrates antioxidant activity in scavenging 1,1-diphenyl-2-picrylhydrazyl (DPPH) radicals and exhibits strong activity similar to vitamin C. Therefore, *C. longa* has the potential to be developed for its anti-aging effects or prevention of premature aging. The antioxidant activity works synergistically to stabilize Reactive Oxygen Species (ROS) during the process of photoaging [103,107]. Many SAD people use *C. longa* as a body scrub and mask for skin care.

According to a study by Lestari U. et al. (2023), the ethanol extract of *T. sinensis* leaves at concentrations of 2.5%, 5%, 7.5%, and 10% was compared to the positive control, vitamin C, using the DPPH method to measure antioxidant activity [52]. The IC_50_ value of the ethanol extract of *T. sinensis* leaves was determined to be 12.351 ppm, while that of vitamin C was 7.805 ppm. These results indicate that the ethanol extract of *T. sinensis* leaves exhibits strong antioxidant activity, with an IC_50_ value below 50 ppm and approaching the IC_50_ value of vitamin C. The antioxidant activity works synergistically to counteract Reactive Oxygen Species (ROS) during the process of photoaging. Therefore, the ethanol extract and active fractions of *T. sinensis* leaves have the potential to be developed into pharmaceutical formulations as anti-aging agents [110].

Another plant with anti-aging activity and bioactive compound content is *C. heyneana*. In a study conducted by Gupta, S. et al. (2024), in vivo testing was performed by observing the histomorphological changes in the skin of rats exposed to ultraviolet (UV) radiation. The results of the in vivo testing showed that the topical application of *C. heyneana* extract on the rat skin was associated with anti-aging activity. This was evidenced by the improvement in histomorphometric parameters of the UV-induced rat skin, such as epidermal thickness, the number of sunburned cells, collagen tissue, fibroblasts, and elastin, which all improved after the application of *C. heyneana* extract [40].

**Figure 9:**
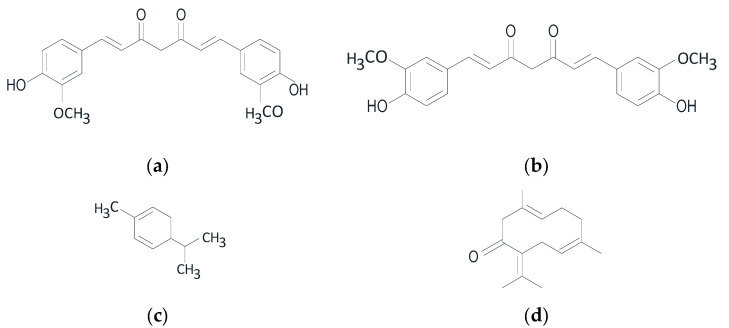
Chemical structures of curcumin (**a**), dihydrocurcumin (**b**), α-phellandrene (**c**), and germacrone (**d**).

The anti-aging activity of four plants from the Zingiberaceae family (i.e., *C. zedoaria*, *K. rotunda*, *C. longa*, and *C. heyneana*) is attributed to bioactive compounds with chemical structures shown in Figure 9 below: curcumin (a), dihydrocurcumin (b), α-phellandrene (c), and germacrone (d). These bioactive compounds exhibit synergistic antioxidant and anti-aging activities [40].

Exposure to UV radiation on rat skin causes dermal changes in cellular components and matrix cells. Free radicals-induced skin damage affects biomolecules, such as lipids, proteins (enzymes), or nucleic acids, triggered by Reactive Oxygen Species (ROS), which leads to the aging process [111]. Reactive Oxygen Species contributes to telomere shortening, autophagy, and cellular fatigue. Oxidative stress significantly influences the age and physiological condition of the body, potentially resulting in degenerative diseases, such as atherosclerosis, insulin resistance, cardiometabolic diseases, neurodegenerative disorders, and premature aging [112]. Therefore, to reduce oxidative stress, antioxidants can be utilized to neutralize free radicals and prevent premature aging. While the body naturally produces antioxidants to inhibit free radicals through cellular oxidation reactions, it also relies on external sources of antioxidants [113].

External antioxidants with potential can be found in *C. zedoaria* and *K. rotunda*, which act as DPPH inhibitors. The results of both activity tests indicate that these plants possess anti-aging properties, making them suitable for development as herbal cosmetics [91]. The aforementioned research yielded results from antioxidant activity tests using the DPPH method. The IC_50_ value of *C. zedoaria* was 2.24 ppm (very strong category), while *K. rotunda* showed a value of 193.17 ppm (weak category). In terms of elastase inhibition, *C. zedoaria* exhibited 49.24% inhibition, whereas *K. rotunda* showed 40.82% inhibition. It can be concluded that *C. zedoaria* exhibits better antioxidant and anti-aging activities compared to *K. rotunda*, making it a suitable ingredient for anti-aging cosmetics [114].

The compound responsible for these anti-aging activities is curcumin (1,7-bis(4-hydroxy-3-methoxyphenyl)-1,6-heptadiene-3,5-dione)—the primary active component found in *C. longa* from the Zingiberaceae family [115,116]. Curcumin compounds have many therapeutic activities, such as antioxidant, anti-inflammatory, and anticarcinogenic. The curcumin compound is the result of isolation and identification that has been carried out on plants belonging to the Curcuma genus, including *Curcuma long* L. (turmeric). Furthermore, there is evidence supporting the potential therapeutic effects of curcumin-rich extracts from *C. longa* in mitigating hepatotoxicity, in addition to its anti-aging effects [117,118].

## 6. Conclusions

Jambi Province, Indonesia is home to the SAD, a minority community residing in the Bukit Dua Belas Forest Area. According to Research on Medicinal Plants and Herbs data, there are 182 plant species used in herbal remedies for skin care. The literature review reveals that only 30 plant species have been identified for their anti-aging properties. Only five plant species are specifically utilized by the SAD community for their skincare treatments. Among the five medicinal plants traditionally used by the SAD in Jambi, Indonesia, *C. longa* has received the most extensive research and shows potential for the development of anti-aging solutions. Its extract and bioactive compound, curcumin, hold promise as new anti-aging agents, both as multi-compound products and single compounds. *C. zedoaria*, *C. heyneana* and *K. rotunda* show good potential for in vivo anti-aging activity. However, the bioactive compounds of these plants need to be evaluated for their in vivo anti-aging effects. *T. sinensis* is the least-studied medicinal plant. Nevertheless, it holds great potential for development, as it is widely used by the SAD community for both traditional medicine and skincare, and this is due to its bioactive compounds that can serve as new anti-aging agents. 

## Figures and Tables

**Table 1:** Indonesian herbal plants acting as antioxidants and anti-aging agents.

No.	Latin Name	Family	Native (Introduced)	Bioactive Phytochemical Compounds	Activity	Bioavailability	Ethnobotany	References
1	*Gnetum gnemon* L.	Gnetaceae	Lesser Sunda Is., Malaya, Maluku, Myanmar, Philippines, Sulawesi, Thailand, Tibet, Vietnam (Cambodia)	Stilbenoid dimer [gnetin C, gnemonoside A, and gnemonoside D] and trans-resveratrol	Anti-aging, anticancer, and antidiabetic	Potential as skin-whitening agent because it contains trans-resveratrol and its derivatives, to inhibit tyrosinase in melanogenesis process	Reduction in uric acid levels	[37]
2	*Curcuma longa* L. and *Curcuma zedoaria* Roxb.	Zingiberaceae	Malaysia, Laos, Myanmar (Burma), Thailand, Vietnam, Brunei, East Timor, Indonesia, Philippines, and Singapore	Terpenoids, flavonoids, and glycosides	Anti-aging, anti-acne, melanogenic, and anti-tyrosinase	Potential anti-aging agent because inhibition of the enzyme elastase and collagenase	Skincare	[38,39]
3	*Curcuma heyneana* Valeton & Zijp	Zingiberaceae	Jawa, Lesser Sunda Is. Indonesia	Curcuminoid (CUR)	Anti-aging, antioxidant, anticancer, and anti-inflammatory	Potentially slowing down aging and/or preventing oxidative stress-induced age-related functional and structural changes and the age-related disorders with result DPPH test yielded 62.5–500 µg/mL and the tyrosinase inhibition and collagenase inhibition tests indicated 31.25–250 µg/mL	Postpartum herbs	[39,40,41]
4	*Pterocarpus indicus* Willd. and *Pterocarpus santalinus* L.f.	Fabaceae	Jawa, Lesser Sunda Is., Malaya, Maluku, Myanmar, Philippines, Sulawesi, Sumatera, Taiwan, Thailand, Vietnam (Andaman is)	Flavonoids, isoflavonoids, terpenoids, phenolic acids, and fatty acids	Anti-inflammatory, anti-aging, antimicrobial, analgesic, and antihyperglycemic	Potential for antioxidant, free radical scavenging	Natural dye	[38,42]
5	*Kaempferia rotunda* L.	Zingiberaceae	Bangladesh, India, Myanmar, Nepal, Taiwan, Thailand, Vietnam, West Himalaya (Jawa, Malaya, Sri Lanka)	Flavonoids, polyphenols, and terpenoids	Anti-aging and antioxidant	showed potent radical-scavenging effects can be shown In the antioxidant activity test, the extracts showed IC_50_ values of 72.61 and 45.75 ppm (strong antioxidant category). In the elastase inhibitor test, the inhibition percentages were 40.82% and 49.24%, respectively (very strong anti-aging category)	Skincare	[43]
6	*Lansium domesticum* Correa	Meliaceae	Borneo, Jawa, Lesser Sunda Is., Malaya, Philippines, Sulawesi, Sumatera, Thailand (Laos, Maluku, Myanmar, Vietnam)	Terpenoids and phenolics	Antimalarial, antifeedant, anti-aging, wound healing, antioxidant, cytotoxic, analgesic, antibacterial, antimutagenic, insecticidal, and larvicidal	Potential as skin-whitening agent and potential anti-aging agent because inhibition of the enzyme elastase and collagenase	Brightening, smoothing, and moisturizing the skin	[43]
7	*Ranunculus blumei* L.	Ranunculaceae	Jawa, Lesser Sunda Is.	Flavonoids, polyphenols, and terpenoids	Anti-aging, antioxidant, and anti-inflammatory	Protective effect on some UVB-induced skin photoaging events such as inflammation, collagen degradation, cellular senescence, skin drying, and melanin production through inhibition of the p38-AP-1	Boosting immune system	[44]
8	*Salacca zalacca* Gaertn.	Arecaceae	Jawa, Sumatera (Borneo, Lesser Sunda Is., Malaya, Maluku, Sulawesi, Vietnam)	Chlorogenic acid	Anti-aging	Have the abilities as antioxidant and anti-inflammatory agent to prevent aging and toxicity because chlorogenic acid exhibits the highest affinity for MMP1, NEP, and PPO3	Boosting immune system	[45]
9	*Intsia bijuga* (Colebr.)	Fabaceae	Bangladesh, India, Jawa, Malaya, Maluku, Philippines, Sri Lanka, Sulawesi, Sumatera, Taiwan, Thailand, Vietnam	Robidanol and robinetin	Anti-aging and antioxidant	Potential for antioxidant, free radical scavenging with DPPH inhibition and antityrosinase enzyme inhibition tests	Anti-inflammatory, treatment for diarrhea, and immune system enhancement	[46]
10	*Glycine max* (L.) Merr.	Fabaceae	China, Korea, Laos, Taiwan, Thailand, Tibet, Vietnam (India, Myanmar, Jawa, Philippines)	Isoflavones (e.g., daidzein)	Anti-aging and antioxidant	The antioxidant and anti-aging potential possessed by black soybeans using H_2_O_2_ scavenging assay indicated an IC_50_ value of 286.24 ± 11.16 µg/mL and hyaluronidase enzyme inhibition test showed an IC_50_ value of 152.56 ± 13.98 µg/mL	Anticancer	[47]
11	*Rhus javanica* L.	Simaroubaceae	India, Jawa, Laos, Lesser Sunda Is., Malaya, Maluku, Myanmar, Sri Lanka, Sulawesi, Sumatera, Taiwan, Thailand, Vietnam	Brusatol and bruceine	Anti-aging	Potential anti-aging exhibited anti-elastase activity with an IC_50_ value of 245.68 μg/mL, and the polyphenol content was 23.28 ± 1.52 mg GAE/g.	Antidiabetic	[48]
12	*Muntingia calabura* L.	Muntingiaceae	Iran, Turkey (Jawa, Lesser Sunda Is, Malaya, Maluku, Sulawesi, Sumatera)	Polyphenols and flavonoids	Anti-inflammatory, antioxidant, and anti-aging	Potential for antioxidant, free radical scavenging	Boosting immune system	[49]
13	*Adenostemma lavenia* L.	Asteraceae	India, Japan, Jawa, Laos, Myanmar, Nepal, Sri Lanka, Sulawesi, Sumatera, Taiwan, Thailand, Tibet	Flavonoids, polyphenols, and terpenoids	Antioxidant, anti-aging, and anti-inflammatory	Potential for antioxidant, free radical scavenging	Boosting immune system	[50]
14	*Myrica javanica* Reinw. ex Bl.	Myricaceae	Borneo, Jawa, Lesser Sunda Is., Philippines, Sulawesi, Sumatera	Phenolic compounds and flavonoids	Anti-aging and antioxidant	Potential anti-aging with inhibitor anti elastase IC_50_ in leaf extract (LE), stem extract (SE), and fruit extract (FE) were found to be 64.71 ppm, 197.49 ppm, and inactive, respectively	Boosting immune system	[51]
15	*Toona sinensis* (Juss.) M.Roem	Meliaceae	Jawa, Laos, Malaya, Myanmar, Nepal, Pakistan, Sri Lanka, Sumatera, Thailand, Tibet, Vietnam	Phenolic compounds and flavonoids	Anti-aging and antioxidant	Potential for antioxidant, free radical scavenging with; the IC_50_ value of the ethanol extract of *Toona sinensis* was 12.351 ppm, while vitamin C was 7.805 ppm	Treating skin diseases	[52]
16	*Syzygium aromaticum* (L.) Merr. & L.M.Perry	Myrtaceae	Maluku	Flavonoids, eugenol, trans-β-caryophyllene, α-humulene, eugenol acetate, caryophyllene oxide and trimethoxy acetophenone	Anti-aging	The potential anti-aging activity, which is based on CLS assay a concentration of 8 µg·mL−1 could maintain the chronological age of cells dermis	Toothache, stomachache, cough	[53]
17	*Phaseolus vulgaris* L.	Fabaceae	Jawa, Philippines, India, Korea, Myanmar, Vietnam, Malaya	Phenolic compounds identified in both crude and purified extracts were quercetin-3-D-galactoside, naringenin, catechin, myricetin, gallic, ferulic and rosmarinic acid	Antimicrobial, anti-inflammatory and ultraviolet radiation (UVR) protective properties	Potential antioxidant and anti-aging with inhibitory against tyrosinase and elastase enzymes	Cardiovascular	[54]
18	*Phyllanthus emblica* L.	Phyllanthaceae	Jawa, Laos, Lesser Sunda Is., Malaya, Myanmar, Pakistan, Sri Lanka, Sumatera, Taiwan, Thailand, Vietnam, India	Phenolics, terpenoids, alkaloids, glycosides, flavonoids, tannins, and saponins	Antioxidant, anti-aging, anti-inflammatory	Potential anti-aging with strong antioxidant showed strong protective effect against the aging process in Caenorhabditis elegans model, including increased thermal resistance, extended lifespan	Boosting immune system	[55]
19	*Echinacea purpurea* (L.) Moench	Asteraceae	Jawa, Korea, Krym, Lesser Sunda Is., Malaya, Maryland, Myanmar, Philippines, Sulawesi, Sumatera	Polysaccharides, flavonoids, caffeic acid derivatives, and essential oils	Antioxidant, anti-aging, anti-inflammatory	Potential anti-aging with activity anti-collagenase, anti-elastase and anti-hyaluronidase activity	Boosting immune system	[56]
20	*Centella asiatica* (L.) Urb	Apiaceae	Jawa, Lesser Sunda Is., Malaya, Maluku, Myanmar, Pakistan, Philippines, Sri Lanka, Sulawesi, Sumatera, Thailand, Vietnam	Phenolic groups such as flavonoids and isoprenoids (terpenoids and saponins. The phenolics include kaempferol and quercetin	Antioxidant, anti-aging, anti-inflammatory	Potential for antioxidant and anti-aging free radical scavenging	Boosting immune system	[57]
21	*Cosmos caudatus* Kunth	Asteraceae	Jawa, Myanmar, Philippines, Sri Lanka, Thailand, Vietnam	Catechin, α-tocopherol, myoinositol, stigmasterol, lycopene, quercetin, quercetin 3-O-arabinofuranoside, quercetin 3-O-rhamnoside, quercetin 3-O-glucoside, quercetin 3-O-xyloside, routine, and chlorogenic acid	Antioxidant, anti-aging, and anti-inflammatory	Potential for antioxidant, free radical scavenging	Blood circulation, strengthens bones, treats burns, muscle tension, spasms, anti-aging, and treats infectious diseases	[58]
22	*Carthamus tinctorius* L.	Asteraceae	Jawa, Laos, Lesser Sunda Is., Malaya, Maluku, Myanmar, Philippines, Sulawesi, Sumatera, Thailand, Tibet, Vietnam	Quinochalcones, flavonoids, alkaloids, polyacetylene, organic acids, and other compounds	Antimicrobial, antimalarial, antiacne and antidandruff, anticancer and antitumor, anti-imunodepressant, anti-inflammatory, anti-inflammatory, antioxidant, and anti-aging	Potential anti-aging in to inhibits collagenase and elastase activity of 72.1% at 500 μg/mL, corresponding to IC_50_ = 130.1 μg/mL. The action of antioxidant activity can reduce skin aging and skin wrinkling	Treating measles	[58]
23	*Spatholobus littoralis* Hassk.	Fabaceae	Borneo, Jawa, Philippines	Catechin, daidzein, formononetin, glycitein, luteolin, apigenin, hesperetin, naringenin, and kaempferide as elastase inhibitors	Anti-aging, photoprotective, antioxidant, and anti-inflammatory	Inhibition of elastase activity can prevent skin aging	Anticancer	[59]
24	*Cinnamomum burmannii* BL	Lauraceae	Jambi, West Sumatra, South Bandung, Indonesia	Cinnamaldehyde and eugenol, trans-cinnamaldehyde, trans-cinnamyl acetate; terpinene: (-)-spathulenol; caryophyllene; D-borneol; eucalyptus; guaiole	Antibacterial, antifungal, antioxidant, anticancer, anti-inflammatory and antidiabetic activities	Potential for antioxidant, anti-inflammatory, and antibacterial properties	Spices	[60]
25	*Piper nigrum* L.	Piperaceae	India, Malaysia, Indonesia, China, Thailand, Sri Lanka, Vietnam, Brazil and Madagascar	Piperine, alkaloids, flavonoids, carotenoids, terpenoids, phenolics and sterols	Antiplatelet, antihypertensive, anticancer, antioxidant, analgesic, antidepressants and anti-diarrheal	Potential for antioxidant with inhibition of free radical formation by scavenging and suppression of degenerative and chronic diseases	cough, cold, dyspnea throat diseases, intermittent fever, dysentery, stomachache, worms and piles	[61]
26	*Zingiber officinale* Roscoe	Zingiberaceae	Lesser Sunda Is., Malaya, Myanmar, Philippines, Sri Lanka, Taiwan, Thailand, Vietnam	Curcuminoids (curcumin, demethoxycurcumin, and bisdemethoxycurcumin)	Anti-aging, anti-inflammatory, anticancer agent and antibiotic	Potential anti-aging based on and in vivo studies and clinical trial results	Spices	[62]
27	*Nephelium lappaceum* L.	Sapindaceae	Malaya, Maluku, Myanmar, Philippines, Sulawesi, Sumatera, Thailand, Vietnam Jawa, Laos, Lesser Sunda Is.	Ellagic acid, corilagin, geraniin, quercetin, and rutin	Anti-aging, antioxidant, anti-inflammatory, antiviral, anticancer, and antibacterial	Anti-ageing biological activities including the tyrosinase inhibition and the anti-melanogenesis, the collagen biosynthesis	Boosting immune system	[62]
28	*Moringa oleifera* Lam.	Moringaceae	Jawa, Laos, Lesser Sunda Is., Myanmar, Thailand, India, Pakistan	Flavonoids, alkaloids, tannins, and vitamin C	Anti-aging and antioxidant	Potential anti-aging and antioxidant through DPPH, FRAP, and reducing power assay	Increase milk production in nursing mothers	[63]
29	*Nigella sativa* L.	Ranunculaceae	Indonesia, India, Myanmar	alkaloids including nigellicine, nigellimine, nigellidine, 17-O-(β-d-glucopyranosyl)-4-O-methylnigellidine, 4-O-methylnigellidine, nigelanoid, nigeglanine, and 4-O-methylnigeglanine	Anti-aging and antioxidant	potential including anti-aging properties associated with modulation of glycation, collagen cross-linking, and collagenase and elastase activities	Boosting immune system	[64]
30	*Citrus bergamia* (Risso) Risso & Poit.	Rutaceae	Indonesia, Bangladesh, India, Laos, Nepal, Vietnam	Phenolic acids, limonoids and flavonoids	Anti-aging and antioxidant	Potential anti-aging with endogenous antioxidant system, including catalase (CAT) and superoxide dis-mutase (SOD)	Boosting immune system	[65]

## Data Availability

Data sharing is not applicable.
